# Melanoma of the dog and cat: consensus and guidelines

**DOI:** 10.3389/fvets.2024.1359426

**Published:** 2024-04-05

**Authors:** Gerry Polton, Juan F. Borrego, Francisco Clemente-Vicario, Craig A. Clifford, Dariusz Jagielski, Martin Kessler, Tetsuya Kobayashi, Didier Lanore, Felisbina L. Queiroga, Annika Tranaeus Rowe, Péter Vajdovich, Philip J. Bergman

**Affiliations:** ^1^North Downs Specialist Referrals, Bletchingley, United Kingdom; ^2^Hospital Aúna Especialidades Veterinarias IVC Evidensia, Paterna, Spain; ^3^La Merced Veterinary Specialists, Alicante, Spain; ^4^Bluepearl, Malvern, PA, United States; ^5^Veterinary Institute, Faculty of Biological and Veterinary Sciences, Nicolaus Copernicus University, Toruń, Poland; ^6^Department of Clinical Oncology, Tierklinik Hofheim, Hofheim, Germany; ^7^Japan Small Animal Cancer Center, Tokorozawa, Japan; ^8^Oncology Unit, Clinique Hopia, Guyancourt, France; ^9^CECAV, University of Trás-os-Montes and Alto Douro, Vila Real, Portugal; ^10^Evidensia Specialist Animal Hospital Strömsholm, Strömsholm, Sweden; ^11^Department of Physiology and Oncology, University of Veterinary Medicine, Budapest, Hungary; ^12^VCA Clinical Studies, Katonah-Bedford Veterinary Center, Bedford Hills, NY, United States

**Keywords:** dog, cat, melanoma, prognosis, consensus, treatment, guidelines

## Abstract

Melanoma of the dog and cat poses a clinical challenge to veterinary practitioners across the globe. As knowledge evolves, so too do clinical practices. However, there remain uncertainties and controversies. There is value for the veterinary community at large in the generation of a contemporary wide-ranging guideline document. The aim of this project was therefore to assimilate the available published knowledge into a single accessible referenced resource and to provide expert clinical guidance to support professional colleagues as they navigate current melanoma challenges and controversies. Melanocytic tumors are common in dogs but rare in cats. The history and clinical signs relate to the anatomic site of the melanoma. Oral and subungual malignant melanomas are the most common malignant types in dogs. While many melanocytic tumors are heavily pigmented, making diagnosis relatively straightforward, melanin pigmentation is variable. A validated clinical stage scheme has been defined for canine oral melanoma. For all other locations and for feline melanoma, TNM-based staging applies. Certain histological characteristics have been shown to bear prognostic significance and can thus prove instructive in clinical decision making. Surgical resection using wide margins is currently the mainstay of therapy for the local control of melanomas, regardless of primary location. Radiotherapy forms an integral part of the management of canine oral melanomas, both as a primary and an adjuvant therapy. Adjuvant immunotherapy or chemotherapy is offered to patients at high risk of developing distant metastasis. Location is the major prognostic factor, although it is not completely predictive of local invasiveness and metastatic potential. There are no specific guidelines regarding referral considerations for dogs with melanoma, as this is likely based on a multitude of factors. The ultimate goal is to provide the best options for patients to extend quality of life and survival, either within the primary care or referral hospital setting.

## Introduction

Tumors of melanin-producing cells in dogs and cats pose a significant challenge to all practitioners, regardless of experience and facilities. There is a broad spectrum of malignancy and, while patterns of behavior exist, tumors comply with these rules with variable veracity. The following basic rule always applies: advanced clinical stage and evidence of elevated proliferation rate correlate with poorer outcomes. No single treatment is consistently best, and all treatment modalities have a potential application. The purpose of this guideline document is to provide a succinct yet comprehensive overview of melanoma management in dogs and cats.

## Incidence and prevalence

Melanocytic tumors are common in dogs (1, 2) [LOE 2c, OEG B]. Malignant melanoma accounts for 70% of all melanin-producing tumors and 7% of all malignant tumors (1, 3) [LOE 2c-3a, OEG B]. Benign forms are called melanocytomas and account for 30% of melanin-producing tumors (1) [LOE 2c, OEG B]. Middle-aged to older dogs and heavily pigmented breeds are more commonly affected (1, 2) [LOE 2c, OEG B]. Scottish terriers, golden retrievers, poodles, dachshunds, and chow-chows are predisposed to oral melanoma (2) [LOE 2c, OEG B]. Schnauzers, rottweilers, Scottish terriers, golden retrievers, and Irish setters are at increased risk of developing subungual melanoma (4, 5) [LOE 2c-4a, OEG B].

Melanomas are most frequently identified in oral (62%), cutaneous (27%), digital (6%), and subungual (4%) sites (1) [LOE 2c, OEG B]. Melanomas of ocular structures, footpads, nasal cavity, gastrointestinal tract, and anal sacs have been reported (2) [LOE 2c, OEG B]. Oral melanoma is the most common oral malignancy in dogs (2) [LOE 2c, OEG B], representing 14.4 to 45.5% of all oral tumors (6) [LOE 3a, OEG C]. Cutaneous melanoma accounts for 0.8 to 2% of all canine skin tumors (6) [LOE 3a, OEG C]. In dogs, ocular melanocytic tumors exhibit distinct phenotypes: tumors of conjunctiva are usually malignant, whereas limbal, iridal, and uveal tumors are predominantly benign (7, 8) [LOE 3a-4c, OEG C].

Melanocytic tumors are rare in cats. They account for less than 1% of all cancer diagnoses (9) [LOE 5, OEG D], 0.8 to 7.0% of all feline skin tumors and < 1% of feline oral tumors (10, 11) [LOE 4b, OEG C]. They occur most commonly in the eye (limbus and intraocular), the haired skin (especially pinna), and the oral cavity (12) [LOE 4b, OEG C]. Melanoma of the nasal planum has been reported (13) [LOE 4c, OEG C]. The typical age of affected cats is 11–13 years (11, 12) [LOE 4b, OEG C]. Melanoma of the pinna has been reported in a younger patient group (median age 7 years) (11) [LOE 4b, OEG C]. No sex or breed predispositions have been reported (11, 12) [LOE 4b, OEG C].

## History and clinical signs of melanoma in dogs

The history and clinical signs relate to the anatomic site of the melanoma.

Oral melanoma arises primarily in the gingiva, lips, tongue, and hard palate (3, 14–16) [LOE 3a-4a, OEG B]. The tumor may be friable and ulcerated. Clinical signs include halitosis, drooling, bleeding from the mouth, dysphagia, and weight loss (3) [LOE 3a, OEG B]. Enlarged draining lymph nodes may or may not be palpable (17) [LOE 4a, OEG C]. The majority of cases are malignant (2, 3, 14) [LOE 2c-4a, OEG B] but it is noteworthy that a population exists with well-differentiated and slowly progressive tumors arising from the mucous membranes of the lip and oral cavity (18) [LOE 4a, OEG C]. There is variation in the degree of pigmentation; some tumors are completely unpigmented (14) [LOE 4a, OEG C].

Melanocytic tumors of the haired skin typically manifest as raised pigmented mass lesions; signs of inflammation are usually absent. Small brown or black masses are the most common; however, the lesions can be large, flat, or wrinkled (2, 19) [LOE 2c-5, OEG B]. Lesions can be multiple. They may be present for a long time. Cutaneous melanomas are commonly phenotypically benign (1, 2, 19, 20) [LOE 2c-5, OEG B]. Whereas melanocytomas are usually solitary, small, pigmented, firm, and freely moveable over deeper structures, malignant melanomas tend to be fast-growing tumors, often ulcerated and pigmented (21) [LOE 4b, OEG C]. Certain sites may be associated with malignancy: for example, the digit, footpad, and scrotum (2, 21) [LOE 2c-4b, OEG B].

Subungual lesions usually present as a swollen painful distal phalanx or a non-healing lesion near the claw. Patients present with lameness or excessive licking of the site (22) [LOE 4c, OEG C]; subungual melanomas frequently metastasize (5, 22, 23) [LOE 2c-4c, OEG B].

Clinical signs of ocular melanoma relate to the structure affected and may include a mass lesion, glaucoma, hyphema, anterior uveitis, epiphora, conjunctival vascular injection, mucopurulent ocular discharge, and/or protrusion of the third eyelid, depending on the site of the lesion (7) [LOE 3a, OEG C].

### Incidence, prevalence, history and clinical signs. Recommendations:

1. Multiple expressions are used to describe tumors of melanocytic origin. It is recommended that clear and transparent terms are used to ensure a common language and comparability between studies. We would like to reinforce the meanings of the following expressions: *Melanoma and Malignant melanoma* have the same meaning and refer to malignant tumors derived from melanocytes; *Melanocytoma* refers to a benign tumors derived from melanocytes; *Melanocytic tumors* refers to tumors derived from melanocytes, regardless of whether they are benign or malignant.
2. Incidence and prevalence: in dogs, there are known breed predispositions to melanoma. Whether prognoses with melanoma differ according to breed is not known. Given the significance of genotype in human melanoma subtypes, this may be an interesting area of future study.
3. In cats, peak age incidence differs between melanomas of the pinna and melanomas of other sites. Although it is reasonable to presume an aetiological difference between melanomas occurring in sun-exposed and non-exposed sites, there is no evidence to suggest a difference in clinical progression or prognosis.

## Biological aspects and genetics

There are two important differences between human and canine melanomas: 1. canine melanoma is not induced by ultraviolet (UV) light; and 2. whereas ocular and cutaneous truncal melanomas are most common malignant forms in humans, in dogs these anatomic forms are typically benign. Instead, oral and subungual malignant melanomas are the most common malignant types in dogs; these are rare but also aggressive in humans (18, 23) [LOE 2b-5, OEG B].

**Figure 1:**
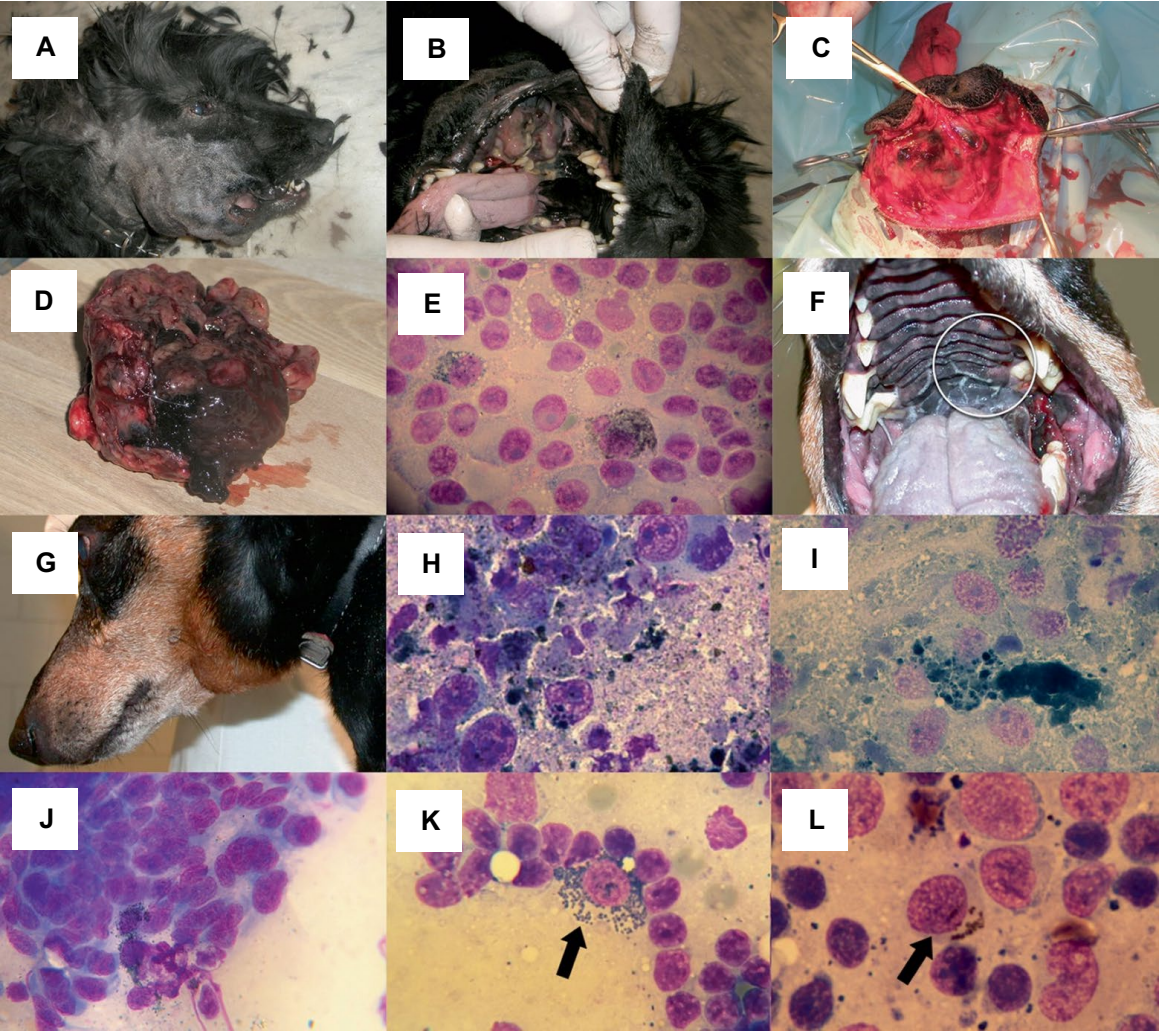
Oral melanoma. **(A–D)** Heterogenous appearance of oral melanoma in a cocker spaniel. **(A)** Outward appearance. **(B)** Visualization of the intraoral aspect illustrates the importance of oral examination in forming the clinical suspicion of melanoma. **(C,D)** The intraoperative and excision specimen images reveal the absence of an appreciable surgical margin. This is acceptable when the goal of surgery is palliation of the consequences of the presence of a large mass or as a precursor to definitive radiotherapy. **(D)** Heterogenous appearance of oral melanoma: the tumor grossly exhibits melanotic and amelanotic parts. **(E)** Malignant melanoma cytology from mandibular gingiva. Cellular borders are usually distinct, though sometimes faint. Cytoplasm is basophilic or light basophilic and granulated; occasionally, small vacuoles or light black to deep green small cytoplasmic granules are seen. Nuclei are eccentrically located and may be irregular; they often show large, dark, prominent nucleoli. **(F–I)** Oral malignant melanoma with lymph node metastasis in a dachshund. **(F)** A small primary tumor was located medial to a left upper molar tooth. **(G)** The patient was presented with left submandibular lymph node enlargement. **(H,I)**. Tumor cells are spindle-shaped and contain large amounts of melanin pigment. The melanoma cells are large and elongated, with length 4-5x and width 2-3x accompanying red blood cells. The cytoplasmic margins are indistinct, the cytoplasm is vacuolated and black/green cytoplasmic granules are apparent. The nuclei are round to oval with stippled, coarse chromatin; nucleoli are distinct and vary in size and number. **(J)** Spindle-shaped form of malignant melanoma cytologically resembles a sarcoma, though melanin granules are also evident in this sample. **(K,L)** Oral melanoma with lymph node metastasis; melanophages versus malignant melanoma. **(K)** A macrophage (melanophage) with intracytoplasmic melanin pigment (arrow) is seen among reactive lymphoid population. Free melanin granules are also visible. Melanophages in themselves do not indicate tumor metastasis, as they can be seen in reactive or normal lymph nodes from dogs without melanocytic tumors. **(L)** Intracytoplasmic melanin pigment is evident in a large epithelioid melanoma cell (arrow) among a population of reactive lymphoid cells. **(E,I,J–L)** Original magnification x 1,000. **(H)** Original magnification x 800.

Melanomas in the oral cavity are similar to human mucosal melanomas in that benign and malignant forms cannot be discriminated by visual examination (3, 24–26) [LOE 2a-5, OEG D]. Pigmentation can vary within a single tumor (Figure 1). Tumor burden and pigmentation are inconsistent indicators of malignant potential.

In humans, *BRAF* mutation plays an important role in the pathogenesis of UV-exposed cutaneous melanoma; there is a low frequency of *BRAF* mutations in dogs, consistent with their differing etiologies (27) [LOE 2b, OEG B]. In canine malignant melanoma and melanocytoma, abnormalities of tumor suppressor expression or localization affecting p16, PTEN, p53, Rb and p21 have been noted, but explicit causal associations with malignant progression are yet to be demonstrated (28) [LOE 4b, OEG A]. In canine malignant forms, there is a gain of chromosomes (CFA) CFA 13 and CFA 17 and loss of CFA 22. Melanocytomas show fewer aberrations but gain locus CFA 20q15.3–17 (29) [LOE 4a, OEG A]. As in human mucosal melanomas, canine mucosal melanomas show a conserved deletion and insertion event on CFA 30 (HSA 15 in human melanoma), gain of c-MYC, and deletion of CDKN2A (30–32) [LOE 4a-5, OEG A]. Investigations into the genetics of canine melanoma have also revealed alterations in CFA 30 and CFA 10, causing MDM2 and CDK4 changes, and mutations on *NRAS*, *KRAS*, *PTEN*, and *TP53* (29, 32, 33) [LOE 4a-4b, OEG A] are noted. *PTPRJ* has been shown to be inactivated in some canine mucosal melanomas (32) [LOE 4b, OEG B].

## Diagnosis

### Cytology

Melanoma subtypes exhibit great variation in their cytomorphology. Cells may mimic round cell, epithelioid, or spindle-shaped (Figure 1) neoplasms. Balloon-cell variants are occasionally seen (19, 25, 34, 35) [LOE 5, OEG D]. Individual neoplastic cells have a round to oval nucleus and prominent, large, pale to dark-staining nucleoli (6, 34, 36) [LOE 5, OEG D]. Anisocytosis and anisokaryosis are frequent. Variable numbers of mitoses can be observed (35, 36) [LOE 5, OEG D]. Inflammatory cell infiltrates will be present if there is tissue necrosis.

While many melanocytic tumors are heavily pigmented, making diagnosis relatively straightforward, melanin pigmentation is variable (35, 36) [LOE 5, OEG D]. The cytoplasmic melanin pigment granules may be fine and dust-like, needle-shaped, or coarse and granular. Small amounts of intracellular gray/black/green pigment may be seen (36) [LOE 5, OEG D]. If the neoplastic cells are disrupted during sample collection or preparation, melanin pigment may be evident in the background of the smear. Infrequently, melanomas may be devoid of melanin pigmentation, rendering cytological diagnosis challenging or impossible (6, 34) [LOE 5, OEG D].

Fine needle aspirates from lymph nodes can be extremely helpful for staging dogs with melanoma, particularly in patients with gross lymphadenomegaly (17) [LOE 4a, OEG A] (Figure 1). However, diagnosis of lymph node metastasis can be challenging due to the varied appearance of metastatic melanocytes and the presence of melanin-containing macrophages (melanophages) (37) [LOE 4b, OEG A]. Dermal melanin is taken up by macrophages, which then traffic to the lymph nodes. Differentiation between melanocytes and melanophages may require histology, immunohistochemistry, or other histochemical staining techniques.

### Biopsy

The identification of neoplastic cells containing melanin pigment allows for a cytologic diagnosis of melanoma in many cases (2, 38) [LOE 5, OEG D]. However, poorly pigmented or amelanotic melanomas are more challenging to diagnose cytologically, and histopathology with or without immunohistochemistry is required to confirm a diagnosis (38) [LOE 5, OEG D].

Differentials for oral melanoma include squamous cell carcinoma, fibrosarcoma, lymphoma, and odontogenic tumors. Cutaneous melanoma can resemble any neoplastic and non-neoplastic skin mass, and for subungual melanoma, SCC and nail-bed infections are differentials (39) [LOE 5, OEG D]. Since oral tumor surfaces, especially those of the large melanocytic tumors, are often ulcerated and/or necrotic, large incisional biopsies, such as deep wedge or core punch biopsies are frequently required to make a definitive diagnosis. For oral tumors, biopsies should only be obtained through the mucosa and not the skin to avoid tumor seeding (40) [LOE 5, OEG D]. Tru-cut biopsy needles can be used, but samples obtained using this method are generally small, making melanin granule detection challenging and potentially limiting a diagnosis to “sarcoma” or “malignant tumor” on histopathology. If Tru-cut biopsies are used, sampling several different areas should be performed. Electrocautery should only be used for hemostasis once the biopsy tissue is removed since biopsy tissues could be damaged by the heat (41) [LOE 5, OEG D]. Finally, all resected tissues should be submitted and properly prepared with an optimized fixation ratio of 1-part tissue to 9-parts 10% neutral buffered formalin (41) [LOE 5, OEG D].

On histopathology, a proliferation of neoplastic cells at dermo-epidermal junctions referred to as “junctional activity,” and neoplastic cells found in intraepithelial nests can aid in the diagnosis of oral melanoma (35, 38) [LOE 5, OEG D]. These features are often observed because of the preservation of epithelium over melanocytic tumors. Thus, if one suspects a poorly pigmented or amelanotic melanoma, a biopsy should be taken, preserving part of the epithelium and improving diagnostic accuracy.

### Histopathology

Specific histochemical staining techniques are employed to demonstrate the presence of melanin: Fontana-Masson (black) and Schmorl’s (blue–green). Bleaching helps visualization of intracellular structures by removing melanin from the tissues. Prussian blue stain aids differentiation of melanin granules from hemosiderin by identifying the presence of iron.

In amelanotic melanoma specimens, immunohistochemistry achieves a definitive diagnosis in almost all cases (19, 37) [LOE 4b-5, OEG B]. Melan-A, melanoma-associated antigen (PNL-2), tyrosine reactive protein (TRP)-1, and TRP-2 are all useful markers. Diagnostic sensitivities of these markers are reported as follows: Melan-A: 81.6%; PNL2: 89.8%; TRP-1: 55.1%; and TRP-2: 79.6%. An immunodiagnostic cocktail comprising all four of these antibodies had 100% specificity and 93.9% sensitivity for identification of canine oral amelanotic melanomas (42) [LOE 4b, OEG B]. For amelanotic spindle cell tumors that lack overlying epithelium, the immunodiagnostic cocktail may still fail to define tumor histogenesis. In that circumstance, RNA expression of TYR, CALD1 and CD34 has been shown to be discriminatory between spindloid oral melanoma and soft tissue sarcoma lesions (43) [LOE 5, OEG D].

The proliferation marker Ki-67 is useful to distinguish benign and malignant forms, and has prognostic value (26, 44, 45) [LOE 4a-4b, OEG A]. A higher percentage of cells express c-kit in melanocytomas than in malignant types (46) [LOE 4b, OEG A], but c-kit expression did not correlate with prognosis in malignant melanomas (46, 47) [LOE 4b, OEG A]. A further summary of prognostically-relevant histological features follows in section: Consideration of Prognostic Indicators and in Table 1.

**Table 1:** Prognostic factors for canine melanoma.

Prognostic factor	Information	Favorable/poor
Anatomic site	Haired skin (non-mucosal) Lip/tongue Oral/digit	Favorable (3, 4, 48) [LOE 3a-4a, OEG B] Favorable (2, 4) [LOE 3a-4a, OEG B] Poor (2–4, 48) [LOE 3a-4a, OEG B]
Stage of disease (1–4)	Prognostic for oral melanoma	Higher stage poor (2, 4, 22, 25, 49) [LOE 3a-4a, OEG B]
Lymph node and distant metastasis	Prognostic for all melanoma	Poor (2, 4, 22, 25, 49) [LOE 3a-4a, OEG B]
Nuclear atypia	Prognostic for all melanoma	Oral: <30% nuclei exhibit atypia, favorable (2, 4, 25, 50, 51) [LOE 3a-4a, OEG B] Other sites: <20% nuclei exhibit atypia, favorable (2, 4, 22, 25) [LOE 3a-4a, OEG B]
Mitotic index	Prognostic for all melanoma	Oral: <4/10 per high power field (hpf), favorable Other sites: <3/10 hpf, favorable (2–4, 25, 26, 45, 48, 50, 51) [LOE 3a-4b, OEG B]
Pigmentation	Prognostic but subjective	Oral: >50% cells pigmented, favorable Other sites: subjectively assessed as highly pigmented, favorable (2, 25, 26, 52) [LOE 3a-4a, OEG B]
Ulceration	Prognostic for cutaneous melanoma only	Poor (25, 26) [LOE 4a, OEG C]
Level of infiltration	Prognostic for all melanoma	Oral: shallow or raised, no bone involvement, favorable (2, 25) [LOE 3a, OEG B] Other: limited to dermis, favorable (2, 25, 48) [LOE 3a-4a, OEG B]
Thickness	Prognostic for non-oral melanoma only	≤0.95-cm tumor thickness, favorable (2, 25, 26, 52, 53) [LOE 3a-4a, OEG B]
Lymphatic invasion	Prognostic for oral melanoma	Poor (2, 4, 25) [LOE 3a-4a, OEG B]
Ki-67	Oral: number of positive nuclei per grid Other sites: % of positive nuclei when 500 cells counted	Oral: <19.5, favorable Other sites: <15%, favorable (2, 25, 26, 45, 52) [LOE 3a-4b, OEG B]
Survivin	Nuclear expression prognostic for cutaneous melanoma only	Poor (26, 54) [LOE 4b, OEG C]

### Biomarkers

In human studies, diagnostic biomarkers currently used to assist in the diagnosis of melanoma are usually specific only for melanocytic neoplasms and not necessarily for their ability to metastasize (55) [LOE 5, OEG D]. In dogs, the high molecular weight melanoma-associated antigen chondroitin sulfate proteoglycan-4 (CSPG4) was found to be a biomarker for malignant melanoma. However, there was no association between CSPG4 staining and clinical stage (56) [LOE 4a, OEG C]. High MicroRNA-126 was prognostic in canine melanoma for a shorter survival time (57) [LOE 4a, OEG C]. For uveal melanomas in dogs, 4 genes demonstrated increased expression in metastasizing compared with non-metastasizing tumors: *HTR2B*, *FXR1*, *LTA4H*, and *CDH1* (58) [LOE 4c, OEG C]. For oral melanomas, a reduced expression of *CXCL12* and an increased expression of *APOBEC3A* was associated with metastasis with classification accuracies of 94% in metastasizing tumors and 86% in non-metastasizing tumors (59) [LOE 4a, OEG C].

Circulating tumor DNA is detectable in the plasma of cancer-affected dogs. By performing droplet digital PCR (ddPCR) or PCR for antigen receptor rearrangement (PARR) methods, tumor-specific point mutations, copy number alterations, and chromosomal rearrangements were detected in cancer-affected dogs, including in 25% of oral malignant melanoma cases in one study (60) [LOE 4b, OEG B].

### Diagnosis. Recommendations:

- A large and/or deep incisional/core biopsy, avoiding ulcerated or necrotic areas, is recommended to make an ACCURATE diagnosis of a melanocytic tumor. This is particularly pertinent in amelanotic or poorly pigmented melanoma.
- Oral melanoma must be biopsied via the mucosal surface, not the skin, to avoid the risk of preventing future curative surgery by iatrogenic tumor seeding.
- If a definitive diagnosis of a suspected melanoma is not made on routine histology, immunohistochemistry is indicated.

### Diagnosis. Opinion:

- In the coming years, it is likely that genomic testing will be able to yield a definitive melanoma diagnosis in cases in which histology and immunohistochemical evaluations have proved non-diagnostic.

## Clinical staging of canine melanomas

Clinical staging of canine oral melanomas is straightforward. The goals are to determine the clinical stage according to the World Health Organization (61) [LOE 5, OEG D] (Box 1), to provide prognostic criteria and to guide therapeutic decision-making. Clinical staging evaluates three classical segments: the primary tumor (size and local extension, T segment), the locoregional lymph nodes (N segment), and the presence of distant metastasis (M segment), mainly in the lungs. A validated clinical stage scheme has been defined for canine oral melanoma. For all other locations and for feline melanoma, TNM-based staging applies.

BOX 1 **Traditional world health organization TNM-based staging scheme for oral melanoma.**

T: Primary tumor (longest diameter)

T1 <2 cm
T2 2-4 cm
T3 >4 cm

N: Locoregional lymph nodes

N0 No evidence of node involvement
N1 Histologic/cytologic evidence of node involvement
N2 Fixed nodes

M: Distant metastasis

M0 No evidence
M1 Evidence

Stage 1 = T1 N0 M0

Stage 2 = T2 N0 M0

Stage 3 = T1 N1 M0 or T2 N1 M0 or T3 N0 M0

Stage 4 = Any T, any N and M1

### Tumor evaluation (segment T)

The size of the local tumor is the first criterion to evaluate. Primary tumor size is of prognostic significance and corresponds to the fundamentals of the TNM classification as follows:

Stage 1: a tumor less than 2 cm in diameter without any metastasis; Stage 2: a tumor greater than 2 and less than 4 cm in diameter without any metastasis; and Stage 3: a tumor greater than 4 cm in diameter and/or with confirmed lymph node involvement.

The tumor size can be clinically measured with a caliper or by cross-sectional imaging with computed tomography (CT). The longest diameter should be used for the classification. Although depth and nature of tissue invasion can be presumed to be prognostically significant, they have not been shown to supersede tumor size and are not included in the recognized clinical stage scheme.

### Locoregional lymph node evaluation (segment N)

Lymph node evaluation is an important step in the clinical staging of dogs with melanoma (2, 4, 22, 25, 49) [LOE 3a-4a, OEG B]. In a study with 100 dogs with oral melanomas, cytological or histopathological evidence of mandibular lymph node metastasis could be demonstrated in 53% of the cases, including in nodes of normal size (17) [LOE 4a, OEG C]. Likewise, subungual melanoma is highly malignant, with 19 to 30% of the dogs reportedly having regional lymph node metastasis at time of diagnosis (22, 62) [LOE 4a-4b, OEG C].

Lymph node size is an unreliable predictor of metastasis (17) [LOE 4a, OEG C]. Similarly, CT (63) [LOE 4c, OEG C] and cytology (37) [LOE 4b, OEG C] have been shown to carry low sensitivity and accuracy for assessment of cervical lymph node metastasis in dogs with oral melanoma. Thus, when lymph node status is uncertain, histopathology should remain the gold standard for the assessment of lymph nodes for metastatic disease with, in some instances, the need for immunohistochemistry (Melan-A) to aid differentiation between melanocytes and melanophages.

In oropharyngeal melanoma, bilateral or contralateral lymph node metastasis is not uncommon. Determination and selective biopsy of the sentinel lymph node is regarded by some to be a more advanced method of staging (37, 64–66) [LOE 4b-4c, OEG C]. However, when an oral tumor is present, the local lymphatic drainage may be abnormal. Metastasis to contralateral lymph nodes and to the retropharyngeal lymph nodes without mandibular lymph node involvement have been described (64, 67, 68) [LOE 4b, OEG B]. Currently, bilateral excisional biopsy of mandibular and retropharyngeal nodes for staging is advocated (62); removal via a single incision has been described (69) [LOE 4b, OEG D]. The parotid lymph node can also be included in the clinical staging. Furthermore, there is not only 1 lymph node per lymphocenter (e.g., dogs can have between 2 and 5 mandibular lymph nodes), and each could be metastatic.

### Distant metastasis (segment M)

Detection of lung metastasis is fundamental in clinical staging of dogs with melanoma. It can be performed by radiography with 3-view thoracic radiographs (70) [LOE 4a, OEG B]. However, CT is more sensitive than radiography for the detection of small pulmonary nodules, especially in large dogs (71–73) [LOE 4b-4c, OEG C]. The lower size threshold for detection is approximately 1 mm by CT, whereas it is between 7 and 9 mm by radiography. In canine cancer generally, only 9% of pulmonary nodules that can be detected by CT are visible on radiographs. In patients with thoracic radiographs without pulmonary nodules, 13 to 39% show nodules on a CT scan (71) [LOE 4c, OEG C].

Abdominal metastases are rare in dogs with melanoma, but have been described in the abdominal lymph nodes, liver, adrenal glands, and other sites (2, 74, 75) [LOE 4a-5, OEG B]. The use of abdominal ultrasonography or CT should be considered. Skeletal metastases are also recognized; clinically occult lesions would not be expected to be identified except by CT.

### Clinical stage. Recommendations:

- Clinical stage determination is fundamental to therapeutic decision-making for canine and feline melanoma.
- We recommend that the size and site of the primary tumor are recorded prior to further intervention to improve communication between colleagues in the event that patient care might be shared.
- Thoracic imaging can be performed by radiography (3-view radiography is preferred), but CT is more sensitive.
- Abdominal metastases are rare but have been described so abdominal imaging would be required to achieve thorough clinical stage determination.

## Melanoma treatment in dogs and cats

### Surgical treatment

Surgical resection using wide margins is currently the mainstay of therapy for the local control of melanomas, regardless of primary location.

### Oral melanoma

In canine oral melanoma, surgical planning using thin-slice contrast CT with multiplanar reconstruction is regarded as the gold standard, especially in all maxillary and caudal mandibular locations. Because resection of melanomas affecting the jaw usually involves a segmental mandibulectomy or maxillectomy, resection width in the bone is based on the detectable bony changes on CT (76) [LOE 4b, OEG B]. For the surgical techniques of jaw resection, please refer to the respective surgical textbooks or monographs.

A minimal surgical margin for melanomas has not been established but, in our experience, a minimum of 1 to 2 cm of healthy-looking bone is usually sufficient to attain clean margins. On the soft tissue side (e.g., lip, sublingual floor of the mouth, soft palate), the surgeon should strive for wider margins (i.e., a minimum of 2 cm). Histopathologic confirmation of completeness of excision is important; all bony and soft tissue margins should be histologically evaluated. There is no consensus among veterinary pathologists regarding surgical margin assessment. Therefore, the veterinarian should discuss with their pathology lab how completeness of resection is assessed and how assessment can be optimized (77, 78) [LOE 3a, OEG D]. Helpful techniques to improve margin assessment include applying surgical ink to the cut surfaces before fixation in formalin or taking resection margin biopsies from bony and soft tissue margins, which are submitted in a separate container. With the latter technique, the surgeon can define which and how many sites are examined histologically. In 70 dogs treated with curative-intent jaw resections (49) [LOE 4a, OEG C], resection of 2 to 3 cm bone margins and 1 cm soft tissue margins resulted in tumor-free margins in almost 73% of cases. Only 17% developed local tumor recurrence. The progression-free interval in this study was 508 days, with a median survival time (MST) of 723 days. In a separate study (79) [LOE 4a, OEG C], almost 80% of resections (73/92) were considered complete after “wide” excision. Recurrence rate was 8.3% (6/73) and the MST was 354 days.

In cats, oral melanoma is rare. A case series of 8 cats treated with radical mandibulectomy reported 6/8 adequately prehending and swallowing food by 3 months after surgery (80) [LOE 4c, OEG C]. In essence, the same resection guidelines apply in cats and dogs, but in cats, less is known about the resection margins necessary to obtain tumor-free margins. Resection width in cats is limited by the much smaller anatomical size of the oral structures. This makes both critical assessment of the resectability of the tumor by appropriate imaging, as well as examination of surgical bony and soft tissue margins, even more important.

### Subungual melanomas

For subungual melanomas, amputation of the affected digit at the metacarpophalangeal or metatarsophalangeal joint is the treatment of choice and usually results in tumor-free margins (81) [LOE 4c, OEG C]. Amputation at the level of the interphalangeal joints is contraindicated because tumor-free soft tissue margins are frequently not achieved. In large tumors, taking resection margin biopsies of the adjacent skin and soft tissue may be indicated to assess completeness of resection.

### Melanoma of the foot pad

Melanoma of the foot pad can be addressed by partial or complete full-thickness resection of the affected foot pad, including the entire fat cushion layer. Assessment of the lateral and deep margins is important, and the use of surgical ink or tumor bed biopsies is recommended. Reconstruction of a weight-bearing foot pad surface can be achieved by transposition of one or both of the second and fifth digital pads using a phalangeal fillet technique (82, 83) [LOE 4c-5, OEG C].

### Melanoma of the haired skin

In non-metastatic melanoma of the haired skin, wide surgical resection is the treatment of choice, whenever anatomically possible. Again, evidence-based recommendations for the resection width have not been established, but most authors recommend a 2 to 3 cm lateral margin and a subfascial resection for the deep extension. Assessment of completeness of resection, using techniques already described, is recommended.

### Ocular melanoma

In ocular melanoma, the location of the tumor on or in the eye is of importance in choosing surgical technique, and outlined as follows (7, 84) [LOE 2a, OEG D]:

1. Conjunctival melanomas in dogs are best treated by surgical resection followed by cryotherapy. If the third eyelid is affected, it should be removed completely. In larger tumors, enucleation is the treatment of choice (7) [LOE 2a, OEG D]. In cats, conjunctival melanomas are highly malignant. Treatment typically comprises orbital exenteration after thorough staging (85) [LOE 4b, OEG C].
2. Limbal melanomas in dogs are frequent and usually affect the dorsal limbus. Most limbal melanomas have a benign behavior, and eye-preserving resection of the tumor by keratectomy/sclerectomy is usually the treatment of choice. Corneal transplant or transplantation of other appropriate tissue (ear cartilage, synthetic material) may be used for reconstruction of large defects. In cases with incomplete resection, adjuvant cryotherapy, photocoagulation, or Strontium-90 usually has a curative effect (86, 87) [LOE 4b, OEG C].
3. Benign iris melanocytomas can be treated successfully by transcorneal laser photocoagulation (7, 84) [LOE 2a, OEG D].
4. Uveal melanomas in dogs and cats are usually locally advanced and therefore best treated by enucleation. The resection margin at the cut edge of the optic nerve should be assessed by histopathology (7, 84) [LOE 2a, OEG D]. A proportion of histologically malignant uveal melanomas in dogs do not progress following complete removal (8) [LOE 4a, OEG C].

### Nasal planum melanoma

In nasal planum melanoma in cats, a wide to radical resection (nosectomy) is recommended for both benign and malignant melanoma. Benign feline nasal planum melanoma may progress to malignancy, and radiation therapy does not result in durable remissions (13) [LOE 4c, OEG C].

Surgical excision of tributary lymph nodes in oral, subungual, and cutaneous melanomas adds important information regarding the clinical stage of the disease and is therefore frequently recommended. However, the therapeutic value of lymph node dissection in canine melanoma has not been studied systematically. Based on the evidence available, lymph node biopsy is more likely a diagnostic rather than a therapeutic procedure and may not offer a survival advantage.

### Radiotherapy

Radiotherapy forms an integral part of the management of canine oral melanomas, both as a primary and an adjuvant therapy (88–95) [LOE 4a-4c, OEG B]. It is also described in the context of feline oral melanomas and in canine non-oral melanoma treatment; although the indications are fewer, responses appear broadly comparable (94, 96–98) [LOE 4a-4c, OEG C].

Although the reason remains controversial, melanoma exhibits sensitivity to coarsely fractionated radiation protocols, with attendant benefits in terms of risk of acute toxicity, costs, and time. This does not mean that melanomas are insensitive to other, more fractionated courses of therapy. An optimal protocol remains undefined. In the context of oral melanoma, it is noteworthy that the canine mandible is a bone that is sensitive to hypofractionation, increasing the risk of osteoradionecrosis (99, 100) [LOE 4c, OEG C]. Lower dose per fraction protocols can be used to reduce late effect incidence in dogs with better prognoses (99) [LOE 5, OEG D].

Treatment protocols reported range from daily 3Gy fractions for 4 weeks (57Gy) through to weekly 10Gy fractions for 3 weeks (30Gy). Most published studies are retrospective (88–90, 95, 96, 101, 102) [LOE 4a-4c, OEG B]. This and the variety of fractionation protocols, equipment used, concomitant therapies, and clinical stage of cancer treated make comparisons between protocols challenging. Outcomes appear to differ between cases that undergo radiotherapy in the macroscopic and the microscopic (post-surgery) settings (89, 93, 94, 101) [LOE 4a-4c, OEG B].

Macroscopic tumors are expected to shrink or disappear in more than 80% of cases; responses become evident within 2 to 3 weeks of initiating therapy. Reported average remission durations range from 3 to 9 months (89, 93, 94, 101) [LOE 4a-4c, OEG B]. Approximately half of all cases experience local recurrence (89, 101) [LOE 4a-4c, OEG C]. Microscopic tumors receiving adjuvant radiotherapy are reported to experience local recurrence in approximately 25% of cases (89) [LOE 4a, OEG C]. Longer remission duration at the primary tumor site is associated with an increasing risk of the development of distant metastasis (90, 101) [LOE 4b-4c, OEG C].

Multiple small studies describe the impact of radiotherapy and chemotherapy in combination. There is an absence of evidence that combining the treatments, with or without surgery, leads to a consistent improvement in outcome (88–90, 101, 102) [LOE 4a-4c, OEG C]. Most studies have failed to demonstrate a positive survival impact of the addition of chemotherapy. However, the small number of cases reported, and a lack of uniformity of tumors and treatments, may mask a genuine difference in outcome. Although statistically significant differences in outcomes may not exist, data from small studies suggested that time to local recurrence was longer in oral melanoma cases receiving adjuvant radiotherapy and chemotherapy in combination compared with adjuvant radiotherapy alone (101) [LOE 4c, OEG C], and time to progression was improved in dogs receiving radiotherapy for melanomas at any site when temozolomide was added to treatment (94) [LOE 4b, OEG C].

There is a general acceptance that technological advances to improve accuracy of radiation targeting will enable greater differentiation between target and non-target tissues, delivery of greater doses of radiation to cancer tissues, and a superior outcome for the patient (103, 104) [LOE 5, OEG D]. Data do not yet exist to substantiate this hypothesis in the field of canine oral melanoma. The complex anatomy of the oral cavity and the infiltrative nature of malignant melanoma apply limits to the improvements that can be gained, but it appears reasonable to believe that further improvements in locoregional melanoma control are possible.

### Medical therapy

#### General principles

While locoregional tumor control can be achieved by means of resection and/or irradiation of the primary tumor and regional lymph nodes, development of metastatic disease very often leads to death. Adjuvant systemic therapy is offered to patients without evidence of macroscopic metastases that are at high risk of developing microscopic disease, and therefore distant metastasis.

### Adjuvant immunotherapy

A therapeutic vaccine containing xenogeneic plasmid DNA with an insert encoding human tyrosinase (ONCEPT® Canine Melanoma Vaccine) has been evaluated in a prospective, multicenter clinical trial in dogs with locally controlled (surgically removed primary tumor, regional lymph nodes, and irradiation for incompletely resected tumors) stage 2 and 3 oral malignant melanoma (105) [LOE 2c, OEG B]. The vaccine was delivered transdermally by use of a needle-free intramuscular vaccination device in 4 doses every 2 weeks, followed by booster injections at 6-month intervals. Statistically significant improvement in survival time was observed (MST not reached; >50% of dogs survived longer than 437 days) versus the classic local-control-only treatment protocol received by a historical control group (MST of 324 days). Another group of vaccinated dogs with stage 1 to 3 oral malignant melanoma reached an MST of 445 days (106) [LOE 4a, OEG C]. Two retrospective clinical studies did not reveal any benefit from the use of the vaccine but, in both studies, local control of the disease was not rigorously achieved. In the first study, less than 35% of patients were treated with surgical margin 1 mm or greater (107) [LOE 4b, OEG C], and in the second, 37% of patients had an incomplete surgical margin (79) [LOE 4c, OEG C]. This reinforces the label recommendation that the vaccine should be used with optimal local disease control (complete surgical margin or radiation therapy if surgical margin was incomplete).

Dogs with digit melanoma treated by digit amputation and a murine tyrosinase DNA vaccine lived significantly longer than those classically treated by digit amputation only (62) [LOE 4a, OEG C]. Significant prognostic factors were presence or lack of metastases at the time of diagnosis (MST of 105 and 533 days, respectively) and stage of the disease (MSTs >952, >1,093, 321, and 76 days for dogs with stage 1, 2, 3, and 4, respectively).

The canine ONCEPT® vaccine can be safely administered to cats with oral, ocular/periorbital, dermal, mucocutaneous, lip, and subcutaneous melanomas. There are no data regarding its efficacy (108) [LOE 4b, OEG C].

### Adjuvant chemotherapy

For canine oral, digital, and cutaneous melanoma, there is no evidence that addition of cytotoxic drugs (carboplatin, cisplatin, melphalan) to surgical treatment or radiotherapy leads to a significant increase in survival time (79, 89, 95, 101, 102, 109) [LOE 4a-c, OEG B]. Cytotoxic drugs (cisplatin 10–30 mg/m^2^ intravenously or carboplatin 90 mg/m^2^ intravenously) may have a role as a radiation sensitizer given once weekly approximately 1 h before radiotherapy in the management of dogs with incompletely excised oral melanomas (90) [LOE 4b, OEG C]. The median time to metastasis was 10.2 months, the MST was 11.9 months, and the local recurrence rate was 15% (90) [LOE 4b, OEG C]. As previously mentioned, adjuvant temozolomide after radiotherapy prolonged time to progression without a significant influence on survival time (94) [LOE 4c, OEG C].

### Treatment of macroscopic disease

Overall response rates for treatment in dogs with measurable disease are disappointing and have not exceeded 18% for dogs treated with cisplatin and piroxicam (110) [LOE 4c, OEG C] or 28% for dogs treated with carboplatin (111) [LOE 4b, OEG C]. Furthermore, complete responses are rare and short in duration. The ONCEPT® vaccine is not indicated for macroscopic disease, but there is information about its use in this setting with an MST of 179 days (106) [LOE 4c, OEG C].

### Other modalities of treatment

Multiple studies have demonstrated a correlation between the expression of COX-2 in canine melanoma and/or melanoma cell lines and proliferation and survival of cells (112, 113) [LOE 2b, OEG B], but no clinical trials investigating nonsteroidal anti-inflammatory drug (NSAID) effectiveness in canine and feline melanoma cases have been published. Local treatment modalities, such as intralesional cisplatin implants or electrochemotherapy with intralesional bleomycin, have been reported for a limited number of patients (114, 115) [LOE 4a-b, OEG C]. Cytotoxic drugs in a metronomic setting could have some influence for immune cells in the microenvironment of a tumor, but the clinical significance of this remains unknown (79) [LOE 4c, OEG C]. Scientific information concerning the treatment of malignant melanoma with tyrosine kinase inhibitors is anecdotal. Masitinib used for stage 3 and 4 malignant melanoma showed only mild effectiveness (116) [LOE 4c, OEG C]. Checkpoint inhibitors successfully used for human melanomas are not currently available for canine and feline patients though the first caninized anti-PD-1 monoclonal antibody has recently been awarded conditional licensure in the United States for treatment of canine melanoma. Gilvetmab was given to 25 dogs with stage 2 or 3 melanoma. Complete response was noted in 2/25, partial response in 3/25 and stable disease in 10/25 cases (117) [LOE 4b, OEG D].

### Treatment. Recommendations:

**Figure 2:**
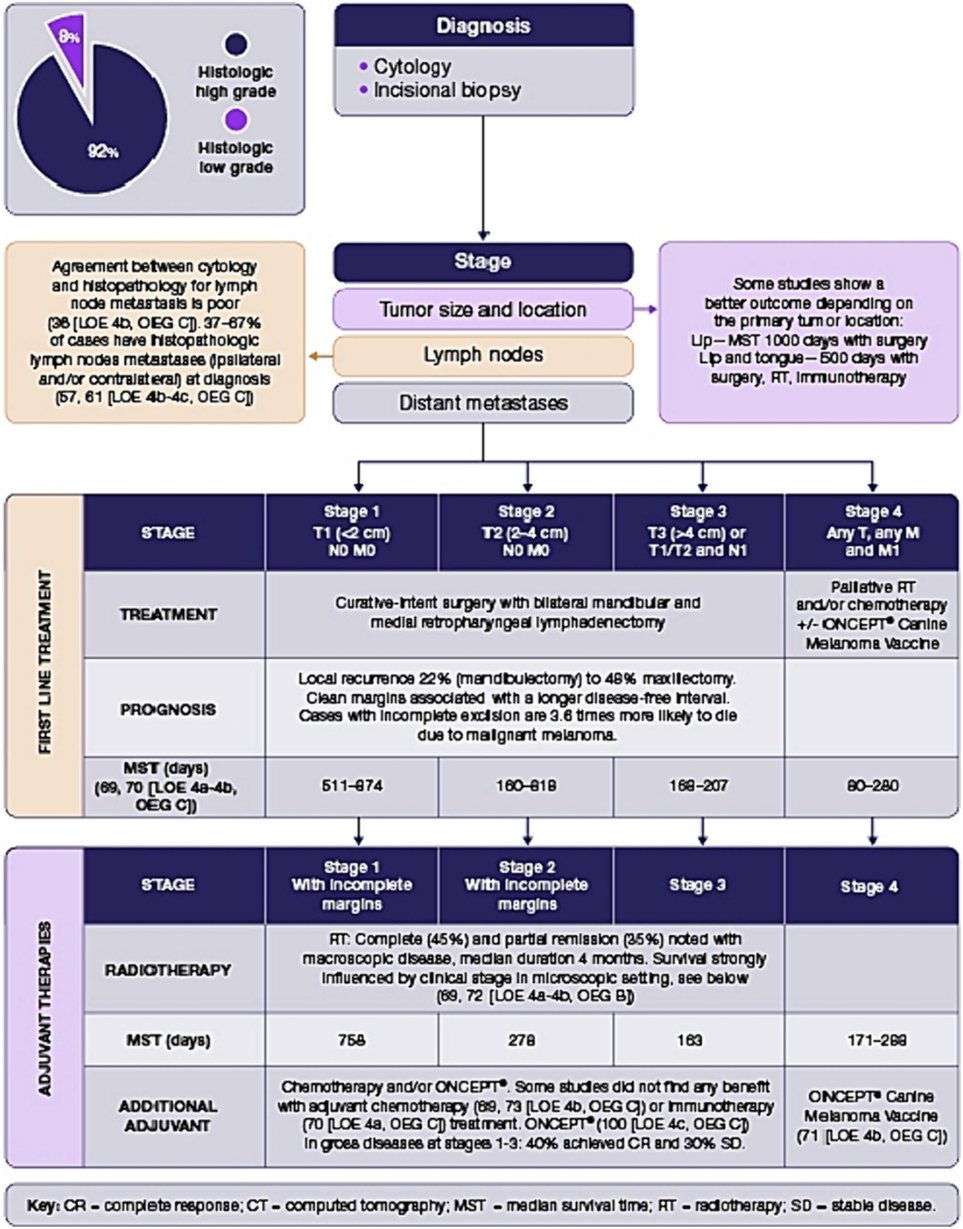
Oral melanoma decision tree.

- Surgical resection using wide margins is currently the mainstay of therapy for the local control of melanomas, regardless of primary location. CT imaging is the optimal strategy for surgical planning of jaw-invasive melanoma.
- Surgical excision of tributary lymph nodes in oral, subungual, footpad and cutaneous melanoma is recommended. It adds important information regarding the clinical stage of the disease and it improves local tumor control.
- When adequate local control cannot be achieved surgically, radiotherapy should be considered.
- Explicit recommendations for treatment of canine oral melanoma are presented in Figure 2.

### Treatment. Opinions:

- An increased array of immunotherapy options is predicted to become available for treatment of melanoma. As a result, radiotherapy is likely to assume greater therapeutic significance because it increases epitope expression.
- Immunotherapy appears to be the optimal method of treating microscopic disease, provided that macroscopic disease is effectively controlled.
- The ONCEPT® Canine Melanoma Vaccine has an evidence base for treatment of stage 2 and 3 malignant melanoma with adequate local tumor control.
- There is limited scientific literature to support the use of other medical therapies for melanoma. A pivotal study of gilvetmab in canine stage 3 melanoma is ongoing at the time of writing.

## Consideration of prognostic indicators

The definition and identification of existing or new prognostic factors in canine melanoma should follow the regimented, systematic approach already described in the literature to clearly identify and consider these factors as truly valuable to the clinician (25, 118) [LOE 2a-3a, OEG C]. Location is the major prognostic factor, although it is not completely predictive of local invasiveness and metastatic potential. The oral/mucosal subtype carries the worst prognosis, with a high degree of local invasiveness and high metastatic potential, and reported MST between 3 and 24 months, according to the stage. Oral benign melanomas might exist (50) [LOE 4a, OEG C]; however, caution is recommended because of their unpredictable behavior (2) [LOE 5, OEG D]. Lip and tongue locations might have a better prognosis compared with other locations within the mouth; well-differentiated mucosal forms have been described (18) [LOE 4a, OEG C]. Melanomas involving the haired skin are often cured with a clean-margin surgical excision (48) [LOE 4a, OEG C]; however, mitotic index of 3/10hpf or higher, high Ki-67 index, and survivin expression seem to be related to metastatic potential and reduced overall survival (26, 52, 54, 119) [LOE 4a-4b, OEG C]. Ulceration might be a negative prognostic factor in cutaneous tumors (26, 52, 81) [LOE 4a-4b, OEG C]. Melanoma in the digits is generally malignant (86%), and all subungual melanocytic tumors are malignant (5) [LOE 2c, OEG B]. They have a high metastatic propensity, with regional or distant pulmonary metastasis evident at the time of diagnosis in 30 to 40% of cases, and subsequent development of regional or distant metastasis in most of the remainder (22, 62) [LOE 4a, OEG B]. Dogs without lymph node or distant metastasis treated by digit amputation have reported median survival times of approximately 12 months, with 1- and 2-year survival rates of 42 to 57% and 11 to 13%, respectively (22, 23, 62, 120) [LOE 4a, OEG B]. Depth of infiltration has been suggested as a negative prognostic indicator for oral melanoma; lack of invasiveness beyond the dermis might carry a favorable prognosis in the cutaneous form (25, 26, 52) [LOE 2-4a, OEG A]. The Ki-67 index is also prognostic for cutaneous and oral melanoma (2, 26, 45) [LOE 2-4b, OEG A] (Table 1).

Following a modified World Health Organization staging system from I to IV, based on tumor size, lymph node involvement, and metastases in dogs with oral melanomas, has been found to be extremely prognostic (49) [LOE 4a, OEG C]. MSTs for dogs with oral melanoma treated with surgery are 511 to 874 days, 160 to 818 days, and 168 to 207 days with stage 1, 2, and 3 disease, respectively (2, 49) [LOE 2-4a, OEG A]. More recent reports suggest dogs with stage 1 oral melanoma treated with standardized therapies, including surgery, radiotherapy, and/or chemotherapy, have an MST of approximately 12 to 14 months, with most dogs dying of distant metastatic disease, not local recurrence (2, 90) [LOE 3a-4b, OEG B].

In cats, tumor location, mitotic index and presence of intratumoral necrosis have been attributed with prognostic significance. A two-tier grading scheme has been proposed, with tumors of the nose, lip and oral cavity considered high-grade if mitotic index is greater than or equal to four OR if there are confluent aggregates of necrotic neoplastic cells. Other non-ocular tumors are considered high-grade if they exhibited BOTH a high mitotic index and intratumoral necrosis (121) [LOE 4a, OEG C]. With so-defined high-grade tumors, median survival time was 90 days whereas only 19% of low-grade tumors adversely impacted survival.

## Melanoma referral considerations

There are no specific guidelines regarding referral considerations for dogs with melanoma, as this is likely based on a multitude of factors. Clinician experience and comfort level, access to diagnostic imaging, and proximity to referral have an impact. Tumor location impacts decision-making, as mandibular and maxillary tumors are more likely to be referred due to the higher level of expertise needed for surgical excision. Access to ONCEPT® Canine Melanoma Vaccine will also affect decision-making as it is limited to selected veterinarians in some geographies and is not available at all in others.

If a referral is to be sought, tumor size should be recorded before excision/referral, as this is deemed a strong prognostic factor. If a biopsy or excision is performed prior to referral, pre-operative digital photographs of the lesion can greatly assist patient assessment and treatment planning. Similarly, staging (imaging and lymph node assessment), when feasible, is paramount before referral, as this may impact the therapy and prognosis. It is also important to note that incomplete excision is associated with poorer outcomes so, if complete excision may not be possible in primary care, referral should be considered.

The ultimate goal is to provide the best options for patients to extend quality of life and survival, either within the primary care or referral hospital setting.

### Referral considerations. Recommendation:

Enrollment of patients into clinical trials will help to address important unanswered clinical questions.

## Author contributions

GP: Writing – review & editing, Writing – original draft, Supervision, Project administration, Methodology, Conceptualization. JB: Writing – review & editing, Writing – original draft. FC-V: Writing – review & editing, Writing – original draft. CC: Writing – review & editing, Writing – original draft. DJ: Writing – review & editing, Writing – original draft. MK: Writing – review & editing, Writing – original draft. TK: Writing – review & editing, Writing – original draft. DL: Writing – review & editing, Writing – original draft. FQ: Writing – review & editing, Writing – original draft. AR: Writing – review & editing, Writing – original draft. PV: Writing – review & editing, Writing – original draft. PB: Writing – review & editing, Writing – original draft.
