# Corrigendum: Melanoma of the dog and cat: consensus and guidelines

**DOI:** 10.3389/fvets.2024.1442751

**Published:** 2024-07-02

**Authors:** Gerry Polton, Juan F. Borrego, Francisco Clemente-Vicario, Craig A. Clifford, Dariusz Jagielski, Martin Kessler, Tetsuya Kobayashi, Didier Lanore, Felisbina L. Queiroga, Annika Tranaeus Rowe, Péter Vajdovich, Philip J. Bergman

**Affiliations:** ^1^North Downs Specialist Referrals, Bletchingley, United Kingdom; ^2^Hospital Aúna Especialidades Veterinarias IVC Evidensia, Paterna, Spain; ^3^La Merced Veterinary Specialists, Alicante, Spain; ^4^Bluepearl, Malvern, PA, United States; ^5^Veterinary Institute, Faculty of Biological and Veterinary Sciences, Nicolaus Copernicus University, Toruń, Poland; ^6^Department of Clinical Oncology, Tierklinik Hofheim, Hofheim, Germany; ^7^Japan Small Animal Cancer Center, Tokorozawa, Japan; ^8^Oncology Unit, Clinique Hopia, Guyancourt, France; ^9^CECAV, University of Trás-os-Montes and Alto Douro, Vila Real, Portugal; ^10^Evidensia Specialist Animal Hospital Strömsholm, Strömsholm, Sweden; ^11^Department of Physiology and Oncology, University of Veterinary Medicine, Budapest, Hungary; ^12^VCA Clinical Studies, Katonah-Bedford Veterinary Center, Bedford Hills, NY, United States

**Keywords:** dog, cat, melanoma, prognosis, consensus, treatment, guidelines

In the published article, there was an error. In the Conflict of Interest section, one author (Philip J. Bergman) forgot to declare a potential conflict of interest.

A correction has been made to the Conflict of Interest statement. This sentence previously stated:

“The authors declare that the research was conducted in the absence of any commercial or financial relationships that could be construed as a potential conflict of interest.”

The corrected sentence appears below:

“PB receives a yearly minority royalty stream payment through Boerhinger Ingelheim Animal Health (BIAH) for a license related to xenogeneic DNA patent #US20020150589A1. He also serves on the BIAH VOAB (Veterinary Oncology Advisory Board) and is a paid speaker on various oncology topics.

The remaining authors declare that the research was conducted in the absence of any commercial or financial relationships that could be construed as a potential conflict of interest.”

The authors apologize for this error and state that this does not change the scientific conclusions of the article in any way. The original article has been updated.

